# A Large-Scale Bank of Organ Donor Bone Marrow and Matched Mesenchymal Stem Cells for Promoting Immunomodulation and Transplant Tolerance

**DOI:** 10.3389/fimmu.2021.622604

**Published:** 2021-02-26

**Authors:** Brian H. Johnstone, Franka Messner, Gerald Brandacher, Erik J. Woods

**Affiliations:** ^1^Ossium Health, Indianapolis, IN, United States; ^2^Department of Biomedical Sciences, College of Osteopathic Medicine, Marian University, Indianapolis, IN, United States; ^3^Department of Plastic and Reconstructive Surgery, Johns Hopkins University School of Medicine, Baltimore, MD, United States; ^4^Department of Visceral, Transplant and Thoracic Surgery, Center of Operative Medicine, Medical University of Innsbruck, Innsbruck, Austria; ^5^Department of Medical and Molecular Genetics, Indiana University School of Medicine, Indianapolis, IN, United States

**Keywords:** immune tolerance, chimerism, bone marrow, vascular composite allograft, regulatory T cells, solid organ transplant, mesenchymal stem cells, hematopoietic stem cell

## Abstract

Induction of immune tolerance for solid organ and vascular composite allografts is the Holy Grail for transplantation medicine. This would obviate the need for life-long immunosuppression which is associated with serious adverse outcomes, such as infections, cancers, and renal failure. Currently the most promising means of tolerance induction is through establishing a mixed chimeric state by transplantation of donor hematopoietic stem cells; however, with the exception of living donor renal transplantation, the mixed chimerism approach has not achieved durable immune tolerance on a large scale in preclinical or clinical trials with other solid organs or vascular composite allotransplants (VCA). Ossium Health has established a bank of cryopreserved bone marrow (BM), termed “hematopoietic progenitor cell (HPC), Marrow,” recovered from deceased organ donor vertebral bodies. This new source for hematopoietic cell transplant will be a valuable resource for treating hematological malignancies as well as for inducing transplant tolerance. In addition, we have discovered and developed a large source of mesenchymal stem (stromal) cells (MSC) tightly associated with the vertebral body bone fragment byproduct of the HPC, Marrow recovery process. Thus, these vertebral bone adherent MSC (vBA-MSC) are matched to the banked BM obtained from each donor, as opposed to third-party MSC, which enhances safety and potentially efficacy. Isolation and characterization of vBA-MSC from over 30 donors has demonstrated that the cells are no different than traditional BM-MSC; however, their abundance is >1,000-fold higher than obtainable from living donor BM aspirates. Based on our own unpublished data as well as reports published by others, MSC facilitate chimerism, especially at limiting hematopoietic stem and progenitor cell (HSPC) numbers and increase safety by controlling and/or preventing graft-vs.-host-disease (GvHD). Thus, vBA-MSC have the potential to facilitate mixed chimerism, promote complementary peripheral immunomodulatory functions and increase safety of BM infusions. Both HPC, Marrow and vBA-MSC have potential use in current VCA and solid organ transplant (SOT) tolerance clinical protocols that are amenable to “delayed tolerance.” Current trials with HPC, Marrow are planned with subsequent phases to include vBA-MSC for tolerance of both VCA and SOT.

## Introduction

SOT has become standard of care over the last half century, resulting in not only a significant life extension but also an enhancement of quality of life (QOL) for end-stage organ failure patients (1, 2). More recently, VCA has become a life changing procedure for patients with severe deformities due to traumatic injury or congenital defects (3). While short-term outcome of transplant recipients using refined conventional immunosuppressive protocols have steadily improved, long-term outcome for the vast majority of patients has not changed over the last decades of experience with transplantation; chronic rejection nearly inevitably leads to organ loss and, depending on the transplanted organ, also to patient death unless a retransplantation is performed (4). Ten years after transplantation only roughly 50% of all heart, liver and kidney and 30% of lung and intestinal grafts are still functioning^1^.

The continuing negative impact of chronic rejection, combined with the severe adverse effects of conventional immunosuppressive regimens, has spurred intense research into new and safer strategies to prevent allograft rejection. While chronic allograft failure is associated with more frequent hospitalization, higher morbidity and increased health care costs, chronic immunosuppression (IS) is linked to side effects that range from malignancy, infection, toxicities (kidney, central nervous system, hematopoietic system) to cardiovascular and metabolic diseases (5–15). Medication related adverse effects, amount of pill-intake, combined with high costs of immunosuppressive drugs, translate to high rates of patient non-compliance/non-adherence. In kidney transplantation, approximately one third of all patients lose their graft due to non-adherence making it one of the leading causes of allograft loss (16–18). Ultimately, the most desirable outcome and often referred to as the “Holy Grail” of transplantation is the establishment of transplant tolerance as this would abrogate the need for chronic IS, thereby transforming organ transplantation from a chronic treatment to a permanent cure (19). Tolerance in the setting of organ and tissue transplantation not only leads to improved QOL, it also eliminates drug-related side effects, mitigates the impact of adherence and compliance, substantially lowers health care cost, extends organ half-life, and thereby addresses the ongoing critical issue of organ shortages (10, 12, 13, 20–26).

Besides tolerance regimens, various alternative strategies to inhibit rejection are in development to replace or reduce the need for current mainstay IS drugs. These regimens seek to shift the balance of lymphocytes in favor of regulatory T (Treg) cells over effector/memory T (Tem) cells, as opposed to pan-T cell inactivation with calcineurin or mTOR inhibition (27, 28). Of particular note is the increasing number of exploratory cell-based immunoregulatory and tolerizing therapies (29–37). One such immunomodulatory protocol that is already in clinical use utilizes unmodified deceased donor-derived BM cell infusion following HLA-mismatched VCA using a Campath-based induction regimen (38). Even though, only extremely low levels of mixed chimerism were induced, the co-infusion of BM cells after VCA allowed for a substantial reduction of maintenance immunosuppression to a single-agent regimen (32). In contrast to tolerance protocols were transient or stable mixed chimerism-mediated Treg cell expansion and central deletion of donor-specific Tem cells are major drivers of tolerance [reviewed by (33, 36)], durable tolerance in the absence of stable mixed chimerism involves contribution of the graft to long-term promotion of donor-specific T cell suppression/depletion (39).

The vascularized BM component of VCA has innate immunomodulatory properties; however, this is not sufficient to fully tolerize recipients to the highly immunogenic skin component of the composite tissue (29–31, 34, 36, 40). Over 120 upper extremity and >40 facial transplants have been performed worldwide with positive outcomes, demonstrating not only the immunological feasibility but also the potential of this revolutionary life-enhancing modality to restore lost functionality to traumatic injury victims (41, 42). Because reconstructive transplantation addresses a life-changing, but not life-saving, health issue, the risks of non-myeloablative conditioning regimens required to promote mixed chimerism are not warranted. Thus, obtaining durable tolerance in the absence of auxiliary mixed chimerism is a challenge inherent to all forms of transplantation but undoubtedly greatest to VCA (43–47).

Although superficially similar in that both approaches administer hematopoietic cells, there are fundamental differences with respect to safety and mechanisms between the mixed chimerism-based approaches that are currently used in clinical trials to promote tolerance in SOT and the immunomodulatory approach in clinical use for VCA. Induction of tolerance through mixed chimerism necessitates non-myeloablative conditioning in the form of irradiation (total body, total lymphoid, or thymic irradiation), cytotoxic agents (e.g., cyclophosphamide, fludarabine) and cell-depleting agents [e.g., ATG, rituximab; (36, 48)]. Lack of conditioning prior to BM infusion in the VCA tolerance protocol limits chimerism to extremely rare transient events. While cytotoxic effects of conditioning are required to induce tolerance, this toxicity limits its use and is responsible for associated side effects (43–47). Lack of conditioning in immunomodulation not only decreases toxic side effects, but also largely prevents GvHD, which is another major safety concern with hematopoietic stem cell transplantation (HSCT) for SOT (49). Additional strategies to augment BM infusion-mediated immunomodulation in the absence of conditioning to promote mixed chimerism are currently being explored as described below.

### Clinical Experience With BM-Derived Products for Inducing Tolerance and Immunomodulation

Currently, there are three U.S. centers (Massachusetts General Hospital (MGH), Stanford University, and Northwestern University) that are investigating clinical protocols for inducing SOT tolerance (50). The protocols have been reviewed in detail elsewhere (36, 46, 47, 51). Each of these protocols uses a whole or fractionated BM-derived cell transplant to induce stable or transient mixed chimerism. In order to induce tolerance, each of these protocols relies on non-myeloablative conditioning to prepare the BM niche for the engraftment of donor-derived stem cells (52). Current successes in clinical trials using these protocols to induce tolerance of SOT through mixed chimerism have been achieved exclusively in the setting of living donor kidney transplantation (39). Most of the current protocols use a preconditioning regime which is implemented days before the transplant; only one regime exists that starts concomitantly with the transplantation. Due to logistic constraints the procedures are presently limited to elective living donor procedures. However, >80% of all transplant recipients receive grafts from deceased donors. Hence, establishing tolerance protocols for deceased donor organ transplantation would greatly expand the number of patients who could potentially benefit from this life-saving procedure.

VCA grafts are invariably from deceased donors, which are also a source of high quality BM obtained from the donor vertebrae that can be cryopreserved for subsequent infusion (32, 53, 54). In the absence of recipient conditioning, the goal of BM infusion following VCA is to augment chimerism inherent to the composite graft. The protocol used at Johns Hopkins to induce immunomodulation in VCA recipients employs cryopreserved BM that is infused 2 weeks after transplantation. The full complement of mechanisms involved in augmentation is not known but at least partially involves supplementation with regulatory cell types and may additively involve alloreactive clonal T cell exhaustion and deletion (55).

### Toward Developing Clinical Delayed Tolerance Protocols

The achievement of immunomodulation with BM infusion that clinically translates into significantly reduced need for IS in VCA demonstrates that (1) harvesting and cryostorage of deceased donor BM is feasible, (2) cryopreserved deceased donor BM can be safely infused, and (3) delaying infusion of previously cryopreserved deceased donor BM over a significant period following VCA still achieves desirable biological effects. This suggests that delayed tolerance with deceased donor SOT may be possible.

Feasibility of delayed BMT for tolerance in SOT in fact has been demonstrated in rodent and non-human primate models of solid organ and vascularized composite allotransplantation (for details see Table 1). These new protocols paved the way for the introduction of the term “delayed tolerance” which have the distinct advantage of allowing for a recovery period to stabilize graft function and enable inflammation resulting from the surgical procedure as well as ischemia reperfusion injury upon revascularization to subside which may enhance tolerance-promoting effects of the BMT. However, the concomitant increased risk due to expansion of alloreactive Tem cells during the interim must be effectively reduced, which appears feasible in non-human primates using an anti-CD8 monoclonal antibody (67, 68). This finding opens up the potential for banking deceased donor BM for future transplantation to promote tolerance in current as well as future SOT recipients.

**Table 1:** Overview on delayed tolerance protocols in small and large animal models of solid organ and vascularized composite allotransplantation.

**Author**	**Organ**	**Year**	**Time delay**	**Conditioning regime**	**Citation**
**NON-HUMAN PRIMATE**					
Yamada et al.	Kidney	2012	4 months	TBI, TI, Atgam, anti-CD154mAb, anti-CD8mAb	(56)
Lee et al.	Kidney	2013	4 months	TBI, TI, Atgam, anti-CD154mAb, LFA3-Ig	(57)
Tonsho et al.	Lung	2015	4 months	TBI, TI, Atgam, anti-CD8mAB, anti-CD154mAb, anti-IL6RmAb	(58)
Tonsho et al.	Heart, Heart and Kidney	2016	4 months	TBI, TI, anti-thymocyte globulin, anti-CD154 mAb, anti-CD8 mAb	(59)
Huh et al.	Heart and Kidney	2017	2 and 4 months	TBI, TI, anti-thymocyte globulin, anti-CD154 mAb, anti-CD8 mAb	(60)
Hotta et al.	Kidney	2018	4 months	TBI, TI, Thymoglobulin, Belatacept	(61)
Oura et al.	Kidney and Islet	2019	4 months	TBI, TI, Atgam, Belatacept, anti-CD40mAb, LFA3-Ig	(62)
Lellouch et al.	VCA	2020	2 and 4 months	TBI, TI, Atgam, anti-CD8mAB, anti-CD154mAb, anti-IL6RmAb	(63)
**MOUSE**					
Guo et al.	VCA	2019	30 days	TBI, anti-Thy1.2Ab, Cyclophosphamide	(64)
**RAT**					
Chen et al.	Kidney, VCA	2012	2 months	TBI, anti-αβTCRmAb, anti-CD8mAb, ALS	(65)
Xie et al.	Liver	2017	4 weeks	TBI, anti-αβTCRmAb	(66)

*Ig, immunoglobulin; mAb, monoclonal antibody; TBI, total body irradiation; TI, thymic irradiation; VCA, vascularized composite allotransplantation*.

### Ethical Considerations of Translation and Commercialization of Cell Products

Cell therapies are a rapidly growing field that have the potential to significantly impact the practice of medicine, not only in the field of transplantation but for a wide range of diseases (69). Despite their immense potential, cell therapies are significantly more complex in their mode of action and due to biological variation and differences in quality of the starting material, not as standardized as other pharmaceutical products (70). In addition, ethical concerns exist regarding cell and tissue sources and especially the use of altruistically donated cells for commercialization. Similar to the US, European regulations make it illegal to buy or sell human cells and tissues. Yet, it is accepted to compensate for reasonable costs that arise for procurement, processing and storage (71, 72). Ethical and safety concern in the early 2000s led to the implementation of regulations by the U.S. Food and Drug Administration and European Medicines Agency regarding cell- and tissue-based products and therapies (73–76). These regulations ensure strict principles of cell and tissue procurement, product development, processing, testing, distribution, and traceability to maintain quality and safety. However, full compliance with all implemented regulations result in significantly increased production costs disqualifying many products that have been produced by single institutions (77).

### Development of a Genetically Diverse Bank of Deceased Organ Donor Bone Marrow

Deceased donor BM represents a large, untapped source of hematopoietic stem and progenitor cells (HSPCs). As has also been demonstrated over the last few decades with cryopreservation of cord blood, it is well-established that BM remains biologically active following long-term cryopreservation (78–81). The larger volumes of HSPCs that can be recovered from a deceased donor compared to aspiration from living donors allow for multiple HSCT procedures or repeat infusions in cases of graft failure. The recovered BM can be precisely packaged, tested for quality, and cryopreserved for subsequent on-demand use. The cryopreserved units can be stored indefinitely (82), with the advantage over living donor registries of having essentially no attrition.

Efforts are currently underway in collaboration with the national Organ Procurement Organization (OPO) network in the U.S. to build the first bank of cryopreserved deceased donor BM. The U.S. OPO network provides an existing refined infrastructure for procuring and transporting bone tissue recovered from deceased donors. Approximately 10,000 deceased donor organs are recovered each year in the U.S., with a further 40,000 donations, yielding approximately 30,000 organs and over a million tissues recovered annually^2^. The high numbers of bones recovered through this system each year supports the inventory required to establish an integrated system of bone procurement, recovery, and transport, linked to BM processing and banking centers. It has been demonstrated that protocols can be developed and enforced to maintain a favorable ischemic environment from the point of bone procurement and recovery, through cross-country shipping, to arrival at a BM processing center (83). Through these efforts, banking of BM product (HPC, Marrow) for transplantation is currently underway.

Protocols for isolation of HPC, Marrow from deceased donor vertebral bodies were based on original work at University of Pittsburgh and optimized at Johns Hopkins University (32, 53, 54). Those protocols formed the basis for the now fully good manufacturing practice (GMP) compliant process that conforms to 21 CFR Part 1271 regulations and is tested for release in a CLIA-certified laboratory using fully validated testing procedures. Certain improvements to the process were made to increase throughput and enhance reproducibility as well as the aforementioned establishment of logistical procedures for recovery and shipment of vertebrae across large geographic regions. Donor eligibility requirements were established to reduce the risk of adventitious agent transmission (health screening and serological testing) as well as health status incompatible with functioning BM. Finally, cryopreservation conditions were optimized and stability validated to ensure functionality of each HPC, Marrow unit released for transplantation. The result is a product with high viability, high colony forming unit potential and the ability to stably engraft irradiated mice following primary and secondary transplants (manuscript in preparation).

### Discovery and Clinical Development of BM Compartment Mesenchymal Stem/Stromal Cells (MSC) Not Recovered in HPC, Marrow

**Figure 1:**
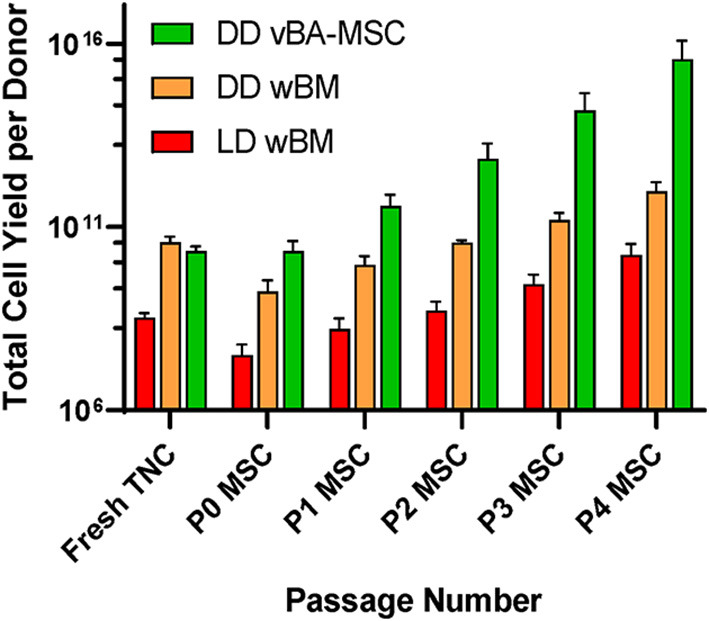
Comparison of total numbers of low passage MSC obtained from living and diseased donor BM. Sources were deceased donor vBA-MSC (DD vBA-MSC) and deceased donor whole BM (DD wBM) and live donor aspirated whole BM (LD wBM). Yields were calculated based on pilot manufacturing runs with either 3 (wBM sources) or 7 (vBA-MSC) donors for each. Averages ± SD shown.

Our team has identified an abundant population of MSC associated with the vertebral body (VB) bone fragment byproduct of HPC, Marrow recovery. These MSC remain tightly adhered to cancellous bone fragments and can only be released by enzymatic treatment. We have determined that these vertebral bone adherent MSC (vBA-MSC) are identical to BM-MSC when cultured (84). The vBA-MSC population yields roughly 2,000x the number of viable, low passage cells from one donor compared with MSC recovered through aspiration from iliac crests of living donors. This bank of vBA-MSC matched to solid organ and VCA donors is a unique resource that overcomes limitations of using third-party MSC by reducing the risk of introducing additional alloantigens and, thus, lowering the risk of sensitization and alloimmune activation (85, 86). Furthermore, the abundance of vBA-MSC allows for generating hundreds of billions of low passage (i.e., P2) cells, allowing multiple infusions (Figure 1).

The potent immunoregulatory properties of MSC comprise a spectrum of secreted and cell-bound molecules that modulate a wide array of innate and adaptive immune responses. The multifaceted mechanisms of MSC immunomodulation have been detailed in numerous reviews and, therefore, will only be briefly introduced here. The MSC secretome includes both freely soluble factors as well as those encapsulated by extracellular vesicles. Mechanisms include metabolic inhibition (e.g., indoleamine-pyrrole 2,3 dioxygenase; IDO), immunomodulatory cytokines (e.g., transforming growth factor-β; TGF-β), and checkpoint inhibitors (e.g., programmed death ligand 1; PD-L1). These myriad factors inhibit T cell activation and proliferation, as well as enhance proliferation of regulatory cells (85–88).

Preclinical studies have demonstrated the therapeutic potential of MSC for inducing operational tolerance of SOT and VCA (89–102), providing proof-of-principle for clinical testing in the transplant setting (100, 103–108). The effect of MSC infusion, including in humans and non-human primates, is to skew the T cell population in favor of Treg over Tem cells (97, 99, 109). Clinical studies of MSC-induced immune tolerance of mismatched kidney transplants have demonstrated safety and efficacy (103–108). In one small study of two kidney transplant patients treated with minimal conditioning and MSC found that levels of CD8+ Tem cells decreased without a decrease in overall T cells (103, 106). Teff cells also demonstrated hyporesponsiveness to alloantigen (110–112). A larger controlled study found significantly higher levels of Tregs at 30 days in the MSC treated cohort compared to the control group (109). Thus, MSC beneficially modulate the ratio of Treg/Tem cells to prevent rejection.

In addition to potentially facilitating graft survival through ameliorating alloreactivity, MSC have demonstrated considerable potential for suppressing GvHD which could be an unintended consequence of SCT to induce tolerance (33, 46, 111, 113–126). In fact, based on a wealth of clinical data, MSC are approved in some countries for the treatment of steroid refractory GvHD and there are strong indications that the cells could be used for prophylaxis (121, 127–131). This potent immunomodulatory function of MSC could mitigate the risk of immune tolerance protocols that promote development of GvHD. However, evidence suggests that this function of the cells is dependent on minimal passaging of the cells, with over-expanded cells losing the ability to modulate acute GvHD (132, 133). The large depot of donor-matched vBA-MSCs facilitates minimal expansion to achieve doses required for treatment in humans (Figure 1).

**Figure 2:**
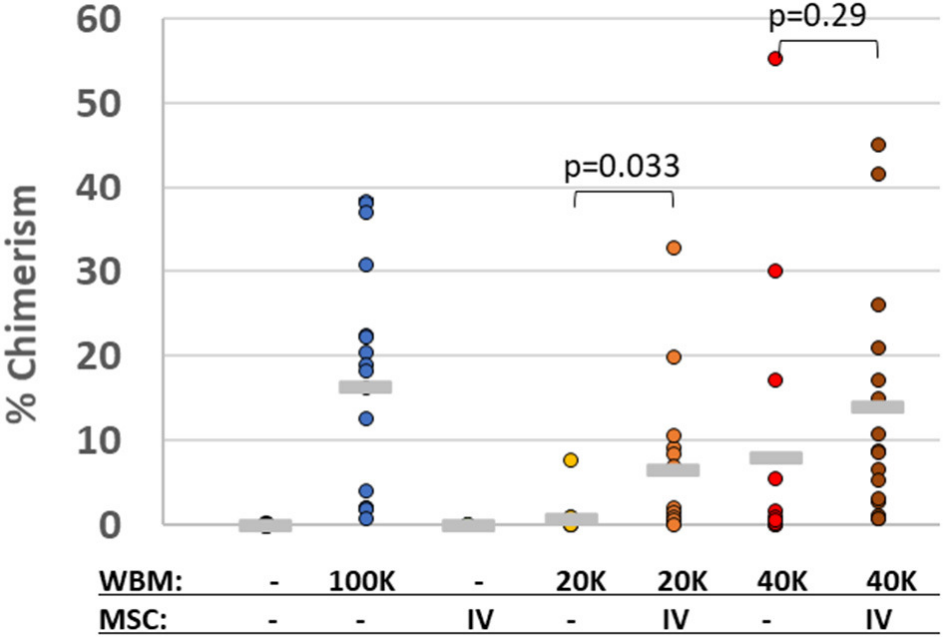
Human MSC promote chimerism of limiting doses of congenic murine bone marrow cells. Lethally gamma-irradiated (875 cGy) C57Bl/6 (CD45.2+) mice were 24 h later with either saline vehicle or one of three doses of whole bone marrow (WBM) isolated from congenic BoyJ (CD45.1+) mice. The WBM doses were either 2 × 10^4^, 4 × 10^4^ or 1 × 10^5^ total nucleated cells (TNC). At 24 and 72 h after irradiation, some groups of mice were also injected with human vBA-MSC (passage 2) at a dose of 1 × 10^6^. Bone marrow was collected from mice surviving 30 days and analyzed for the level of chimerism by flow cytometric analysis with antibodies specific for CD45.1 and CD45.2 surface proteins. The percentage of CD45.1+ chimerism for individual mice in each group is indicated as well as the average per group (horizontal gray line). *P*-values were determined by Student's *t*-Test.

Another mechanism to minimize the risk of GvHD is titrating down the HSPC graft to a minimal efficacious dose, which correspondingly reduces the donor T cell load. MSCs have been reported to facilitate and enhance engraftment of allogeneic HSPC clinically, even after initial graft failure and rejection of conventional stem-cell grafts (134). Preclinical studies suggest that MSCs enhance mixed chimerism when co-infused with HSPC (135) by migrating to the BM stroma to help establish a favorable micro-environment within the hematopoietic niche (136). This appears to minimize the number of HSPC required for transplantation (137, 138). We have confirmed these findings with vBA-MSC in irradiated non-immunocompromised mice treated with limiting dilutions of congenic whole BM with and without co-infusion, followed by a second dose at 48 h, of human vBA-MSC (Figure 2).

Besides GvHD, engraftment syndrome (ES) that occurs in 7–90% of cases during neutrophil recovery after autologous and allogeneic HSCT poses a potential limitation (139). It is associated with fever, pulmonary vascular leak, rash, and organ dysfunction and has also been described in combined HLA-haploidentical BM and kidney transplant recipients. In the described cases, ES manifested not only with fever and fluid retentions but also with a marked acute kidney injury (140), prompting speculations on an increased susceptibility with freshly transplanted kidney grafts, especially in combination with CNI treatment (141). Even though the exact pathophysiology is unclear, ES is thought to be mediated by endothelial cell injury, activated leukocytes, and proinflammatory cytokines. The inflammatory nature of ES is underlined by the good response to treatment with corticosteroids (141, 142). As vBA-MSCs have strong anti-inflammatory, antioxidative, and immunomodulatory properties, co-administration could potentially mitigate the risk or severity of ES after HSCT (143–145). Thus, the combination of promoting BM chimerism and the immunomodulatory functions of MSC suggest that their use as an adjuvant to BM transplants will safely enhance induction of immune tolerance.

### Potential for Incorporating Deceased Donor BM and vBA-MSC Into Current Tolerance Protocols

Each of the current protocols for inducing tolerance in VCA and SOT lend themselves to deceased donor BM augmentation and BMT, respectively, with modification to accommodate donor availability. Inclusion of vBA-MSC either prophylactically or to treat GvHD is possible. Following BM isolation and quality control testing, HPC, Marrow would be cryogenically preserved until shipping under the same conditions for infusion into the patient 14 days following surgery, as described previously (32). Simultaneously, vBA-MSC could be prepared from the bone fragments and expanded before cryopreservation and shipping with HPC, Marrow.

In regard to SOT, the MGH delayed tolerance protocol appears to be the most easily adaptable to HPC, Marrow, providing that encouraging results in NHP and early clinical trials in humans are repeated in future larger clinical trials (56–58, 61, 62, 68, 146–148). Transplantation at 4 months following SOT would allow more than enough time to prepare, qualify and store HPC, Marrow as well as expanded vBA-MSC. It is well-established that cryopreservation preserves cellular function for decades so long as proper controls are implemented to prevent transient warming events (82).

The Stanford protocol, which relies on an infusion of a mixture of isolated mobilized peripheral blood-derived CD34+ and T cells could in theory be adapted to using HPC, Marrow for selection of these cells (49, 149). The amount of HPC, Marrow typically recovered from a full donor contains hundreds of millions of CD34+ cell (53, 54, 83). We have adapted CD34 selection methods to develop a GMP process that has yielded an average of 125 × 10^6^ CD34^+^ cells from HPC, Marrow recovered from three donors. Importantly, these methods can be used on either freshly isolated or previously cryopreserved HPC, Marrow; thus, providing flexibility in cell production. The Stanford protocol infuses cryopreserved selected cells at 11 days after kidney transplant which would provide sufficient time to prepare HPC, Marrow as well as over a billion very early passage GMP vBA-MSC (Figure 1). Given that MSC are commonly dosed at 1 × 10^6^/kg, this would provide more than adequate vBA-MSC for co-infusion as well as any additional dosing if further expansion was not feasible. A company, Medeor, has been established to demonstrate commercial potential of the Stanford protocol and, according to their website^3^, a delayed tolerance protocol for living donor kidney transplantation is in development. As of yet, efficacy has not been established using this protocol with deceased donor kidney transplants.

The Northwestern tolerance protocol for kidney transplantation differs by the use of full body non-myeloablative conditioning with the goal of promoting full chimerism rather than transient (i.e., MGH protocol) or durable (i.e., Stanford protocol) mixed chimerism (150–154). The protocol uses an engineered cell source, termed facilitating cells (FC), derived from kidney donor mobilized blood collected at least 2 weeks prior to transplant combined with HSPC to promote chimerism (155, 156). Providing the protocol is amenable to a delayed tolerance approach, deceased donor HPC, Marrow could offer a distinct advantage for manufacture of FC given the high abundance of BM cells available and the enhanced time provided for manufacture and testing. To this end, we have demonstrated that HPC, Marrow is amenable to manipulation using a CliniMACS system (Miltenyi Biotec). As GvHD appears to be a concern with this protocol, infusion of low passage vBA-MSC could be advantageous.

### Limitations of Current Tolerance Protocols

Despite clinical realization of tolerance and preclinical evidence supporting the feasibility of delayed tolerance protocols as outlined above, tolerance induction is still limited to a few highly specialized centers (47). Widespread adoption is currently hindered by the risks associated with complex recipient conditioning regimes which have a variety of toxic side effects. The most promising strategy of tolerance induction thus far is the mixed chimerism approach, however, tolerance induction efficacy is still limited. HSCT is also associated with a risk of GvHD, which has been observed in protocols aiming for durable chimerism in a small number of patients (36). To overcome these hurdles, a concerted effort of clinicians, scientists, stakeholders (e.g., insurance companies and hospitals), and funding agencies is crucial. In recent years, transplant tolerance has regained attention and a variety of new agents have been identified that have the potential to make induction regimens significantly less toxic, reduce associated risks of GvHD, and increase efficacy. The realization of a deceased bone marrow bank, as outlined in this review, is another step in the process of making transplant tolerance a clinical reality for a larger number of patients.

### Conclusions and Future Perspectives

Future broad success with BM and MSC induction of tolerance and potent immunoregulation will have profound effects on transplant patients. Achieving immune tolerance, in particular, will alleviate the burden of life-long IS and associated morbidity, avoid chronic rejection, and significantly improve overall outcomes. Furthermore, it will overcome current compliance and adherence-based limitations that negatively impact graft survival due to subsequent subliminal rejection and the development of donor-specific antibodies. Tolerance and immunoregulation will be especially impactful for patients by increasing accessibility to transplantation and through positively shifting the risk:benefit ratio by reducing associated long-term risk.

In view of these potential opportunities and substantial benefits, the establishment of a bone marrow bank for delayed tolerance protocols marks a crucial step in making this resource available for present as well as future transplant patients. The complementary treatment with vBA-MSC could further increase safety with the added potential of enhanced efficacy. Furthermore, the ability of vBA-MSC to promote HSPC BM engraftment would allow lowering of HPC, Marrow doses which effectively extends the number of patients who receive organs from a single donor that are able to benefit from this procedure.

## Author Contributions

All authors contributed to writing and editing the article, and approved the submitted version.

## Conflict of Interest

BJ, GB, and EW have equity ownership in Ossium Health. BJ and EW are inventors on patents pending relevant to the subject matter. EW is a co-founder of Ossium Health. The remaining author declares that the research was conducted in the absence of any commercial or financial relationships that could be construed as a potential conflict of interest.

## Footnotes

^1^UNOS https://unos.org/data/transplant-trends/ (accessed October 27, 2020)

^2^unos.org/data/transplant-trends/ (accessed October 27, 2020).

^3^https://www.medeortx.com/our-pipeline.php/ (accessed October 27, 2020).
